# Protosappanin‐A and oleanolic acid protect injured podocytes from apoptosis through inhibition of AKT‐mTOR signaling

**DOI:** 10.1002/cbin.11218

**Published:** 2019-09-08

**Authors:** Jiaxin Zheng, Shoulin Zhang, Huijun Chen, Xiaojun Cai, Chunjian Zhang, Shuhua Li, Yabin Zhou, Jing Shang, Shunyu Liang, Fengzhen Yao

**Affiliations:** ^1^ Department of Nephrology Heilongjiang Academy of Traditional Chinese Medicine Harbin Heilongjiang 150036 PR China; ^2^ Department of Nephrology The Affiliated Hospital to Changchun University of Chinese Medicine Changchun Jilin 130021 PR China; ^3^ Department of Cardiology The Second Hospital Affiliated Heilongjiang University of Traditional Chinese Medicine Harbin Heilongjiang 150009 PR China; ^4^ Department of Nephrology Heilongjiang University of Traditional Chinese Medicine Harbin Heilongjiang 150040 PR China; ^5^ Department of Cardiology The First Hospital Affiliated Heilongjiang University of Traditional Chinese Medicine Harbin Heilongjiang 150040 PR China

**Keywords:** AKT‐mTOR pathway, apoptosis/proliferation, LY294002/IGF‐1, podocyte injury, protosappanin‐A/oleanolic acid

## Abstract

Protosappanin‐A (PrA) and oleanolic acid (OA), which are important effective ingredients isolated from *Caesalpinia sappan* L., exhibit therapeutic potential in multiple diseases. This study focused on exploring the mechanisms of PrA and OA function in podocyte injury. An in vitro model of podocyte injury was induced by the sC5b‐9 complex and assays such as cell viability, apoptosis, immunofluorescence, quantitative real‐time polymerase chain reaction, and western blot were performed to further investigate the effects and mechanisms of PrA and OA in podocyte injury. The models of podocyte injury were verified to be successful as seen through significantly decreased levels of nephrin, podocin, and CD2AP and increased level of desmin. The sC5b‐9‐induced podocyte apoptosis was inhibited in injured podocytes treated with PrA and OA, accompanied by increased protein levels of nephrin, podocin, CD2AP, and Bcl2 and decreased levels of desmin and Bax. The p‐AKT/p‐mTOR levels were also reduced by treatment of PrA and OA while AKT/mTOR was unaltered. Further, the effects of PrA and OA on injured podocytes were similar to that of LY294002 (a PI3K‐AKT inhibitor). PrA and OA were also seen to inhibit podocyte apoptosis and p‐AKT/p‐mTOR levels induced by IGF‐1 (a PI3K‐AKT activator). Our data demonstrate that PrA and OA can protect podocytes from injury or apoptosis, which may occur through inhibition of the abnormal activation of AKT‐mTOR signaling.

**Abbreviations**
CD2AP: CD2‐associated protein
DN: diabetic nephropathy
FITC: fluorescein isothiocyanate
FPs: foot processes
GFB: glomerular filtration barrier
IF: intermediate filament
MN: membranous nephropathy
NHS: normal human serum
OA: oleanolic acid
PI: propidium iodide
PrA: protosappanin‐A
SD: slit diaphragms

## Introduction

Podocytes, highly differentiated glomerular visceral epithelial cells, are important in glomerular filtration barrier (GFB) function. Generally, the major processes and foot processes (FPs) of podocytes are interlinked by slit diaphragm (SD) molecules (Shankland, 1999; Guan et al., 2004). Podocyte injury and loss are an initiating cause of numerous renal diseases. Loss or injury of podocytes leads to proteinuria, which is the major risk factor for the progression of end‐stage renal disease (Hemmelgarn et al., 2010). Therefore, injury to podocytes is a central event in the development of multiple diseases including membranous nephropathy (MN) and diabetic nephropathy (DN) (Durvasula and Shankland, 2006; Shankland, 2006). Currently, several drugs such as rapamycin and triptolide are reported to have therapeutic potential for podocyte injury. However, the limitations of currently available drugs highlight the need for further studies in podocyte injury.

Research has shown that cytoskeletal structure and intercellular junctions are disrupted when podocytes are injured, which is accompanied by decreased levels of nephrin, podocin and CD2‐associated protein (CD2AP), and increased level of desmin (Machado et al., 2012). Nephrin usually functions as an intracellular signaling scaffold in podocytes. Podocin, is a supporting protein that maintains SD integrity and podocyte function (Kang et al., 2010), and is capable of modulating nephrin‐signaling activity by binding to the cytoplasmic tail of nephrin (Boute et al., 2000; Huber et al., 2001; Schwarz et al., 2001; Roselli et al., 2002). CD2AP participates in congenital nephritic syndrome through its C‐terminus interaction with podocin and nephrin (Li et al., 2000; Shih et al., 2001). Finally, CD2AP is also involved with various signaling molecules and assembly of cytoskeleton through its SH3 region (Patrakka et al., 2000; Kim et al., 2003; Wolf and Stahl, 2003). Podocytes were also found to respond to injury by altering their intermediate filament (IF) proteins such as desmin, which is enhanced in podocytes of glomerulosclerosis and DN (Floege et al., 1997; Hoshi et al., 2002). Furthermore, signaling pathways including phosphoinositide 3‐kinase (PI3K)‐AKT, ROS‐NF‐κB, and Wnt/β‐catenin are also implicated in podocyte injury (Wang et al., 2011; Wei et al., 2015). The PI3K‐AKT pathway is well‐known to be the major signaling cascade regulating glucose metabolism, and activation of AKT regulates various processes involved in cancer, such as cell‐cycle progression and growth (Hong et al., 2016). It is suggested that nephrin and CD2AP, together with podocin, interacts with PI3K, and further stimulates PI3K‐dependent, serine‐threonine kinase AKT signaling (Huber et al., 2003). Activation of PI3K‐AKT signaling is also reported to participate in podocyte injury and apoptosis (Chuang and He, 2009; Zhang et al., 2016). Mammalian target‐of‐rapamycin (mTOR) is a key protein kinase downstream of PI3K‐AKT. Through inhibition of mTOR‐ULK1 signaling, rapamycin decreases podocyte injury (Lingling, 2013; Wu et al., 2013). Thus, the PI3K‐AKT‐mTOR signaling pathway is essential for the regulation of podocyte injury. *Caesalpinia sappan* L., a Chinese herb, belongs to the leguminous plant family. Studies have reported that *C. sappan* L. exhibits various biological activities and therapeutic potential for multiple diseases including diabetic complications, burning sensations, and dysentery (Baek et al., 2000; Badami et al., 2003; Kim et al., 2005). Protosappanin‐A (PrA) is an important active ingredient isolated from an ethanol extract of *C. sappan* L. (Wu et al., 2008), and oleanolic acid (OA) is a pentacyclic triterpenoid compound that exists widely in food, medicinal herbs, and other plants (Liu, 1995; Pollier and Goossens, 2012). PrA has an immunosuppressive activity and is essential in the success and survival of transplanted hearts (Wu et al., 2008). In addition, PrA has been proven to inhibit CD4^+^/CD8^+^ ratios of peripheral T cells as well as expression of perforin and granzyme B (Wu et al., 2008, 2010). A study has also reported that OA could prevent the progression of DN induced by streptozotocin (Dubey et al., 2013). However, the effects of PrA and OA on podocyte injury remain unclear.

Previous studies have stated the suitable concentrations of C5b‐6 in vitro model. Assembly and regulation of the membrane attack complex (MAC) were based on structures of C5b‐6 and sC5b‐9, and in vitro concentrations relevance for the human in vivo situation has been suggested (Aleshin et al., 2012; Hadders et al., 2012). C5b‐6 complex can combine with C7, C8, and multiple C9 molecules to construct the sublytic C5b‐9 (sC5b‐9) complex. Chen et al. (2010) used 0.8 µg/mL of C5b‐6, maintaining fixed concentrations of C7, C8, and C9 (10 µg/mL) to assemble sC5b‐9, which established injured podocyte model. sC5b‐9, a macromolecule complex, also known as MAC, can be inserted into the phospholipids bilayer membrane, forming a channel or leaking patch, thus, making the non‐nuclear cells lethal, but nucleated cells such as podocytes will cause no fatal damage (Ma et al., 2013). Here, C5b‐6 complex and normal human serum (NHS) were used to generate sC5b‐9 complex to induce podocyte injury in vitro.

In this study, after podocytes were injured by the sC5b‐9 complex, podocyte apoptosis was increased accompanied by decreased levels of nephrin, podocin, and CD2AP and increased level of desmin. However, treatment of PrA and OA protected podocytes from sC5b‐9‐induced apoptosis, and treatment with both drugs inhibited apoptosis to a significantly greater degree than treatment with any one drug alone. Furthermore, high levels of p‐AKT and p‐mTOR induced by sC5b‐9 were significantly reduced by PrA and OA treatment, and were accompanied by altered expression of several associated genes. These data demonstrated the protective effect of PrA and OA on sC5b‐9 induced‐apoptosis, which may be via regulation of AKT‐mTOR signaling.

## Materials and methods

### Cell culture

MPC5, a mouse podocyte cell line derived from kidneys, was obtained from the Biotechnology Co., Ltd. Shanghai Enzyme Research (Shanghai, China) and cultured in a 5% CO_2_ incubator (Thermo Forma 3111; Thermo Fisher Scientific, Inc., Waltham, MA, USA) at 33°C with RPMI‐1640 (SH30809.01B; Hyclone) medium containing 10% fetal bovine serum (16000‐044; Gibco), 100 U/mL penicillin and 100 mg/mL streptomycin (Invitrogen; Thermo Fisher Scientific, Inc., Waltham, MA, USA), and 10 U/mL IFN‐ɣ (315‐05; Peprotech). A period of time later, cells were passaged and cultured with RPMI‐1640 medium containing 10% fetal bovine serum and 1% double‐antibiotic without interferon γ in a 5% CO_2_ incubator at 37°C for 10–14 days to induce cell differentiation. The medium was replaced daily or every other day according to the growth of the cells.

### Preparation of rabbit anti‐mouse podocyte polyclonal antibody

Nearly, 2 × 10^7^ mouse podocytes were collected, resuspended in 0.5 mL phosphate‐buffered saline (PBS), and sonicated in an ice bath (200w, ultrasound 3 s, and interval 10 s, repeated 30 times). The cell lysate was mixed with Freund's complete adjuvant (FCA) in a 1:1 ratio by volume and emulsified, followed by subcutaneous injections on the skin around the shoulders of the rabbit with 0.1 mL per point. Two weeks later, immunity was strengthened, and the same cell lysate was mixed with Freund's incomplete adjuvant (FIA) in a 1:1 ratio by volume and emulsified, followed by injection subcutaneously with 0.1 mL per point. The immunity was strengthened again every 2 weeks for three more times. One week after the last immunization, blood was collected from the ear artery, and the isolated serum was rabbit anti‐mouse podocyte polyclonal antibody.

### In vitro model of podocyte injury induced by the C5b‐9 complex

Models of podocyte injury were established using the C5b‐6 complex and NHS as a complement source. The venous blood collected from the healthy volunteers were incubated at room temperature for 1 h and then centrifuged at 3,000 rpm, 4°C for 15 min to obtain the supernatant. After 24 h treatment of serum‐free RPMI‐1640 medium, differentiated podocytes were sensitized by incubation with rabbit anti‐mouse podocyte polyclonal antibody for 1 h at 37°C. Subsequently, cells were washed twice with serum‐free RPMI‐1640 medium, and podocytes were incubated with NHS (NHS: serum‐free RPMI‐1640 medium = 1:80) and C5b‐6 complex (204906‐50UGCN; Merck) for 1 h at 37°C.

### Experimental grouping

Different concentrations of C5b‐6 (0.1, 0.2, 0.4, 0.8 µg/mL) and NHS were used to generate sC5b‐9 to induce the model of podocyte injury. After generation of the models, groups generated with low sC5b‐9 (0.1 µg/mL) and High C5b‐9 (0.8 µg/mL) were continually cultured with a normal medium for 48 h, while podocytes cultured in only the presence of medium was regarded as blank. Cells were subjected to cell apoptosis assays, western blot analysis, and immunofluorescence. Models obtained with 0.8 µg/mL C5b‐9 were chosen for treatment with PrA (102036‐28‐2; Biopurify) and OA (42515; Sigma) at different concentrations (0, 10, 20, 40, 80, 160 µM). Podocytes cultured in medium without PrA and OA were considered blank. Models obtained with 0.8 µg/mL C5b‐9 were also randomly treated with either medium; 80 µM PrA; 80 µM OA; 80 µM PrA combined 80 µM OA; or 5 µM LY294002 (a PI3K‐AKT inhibitor, S1105; Selleck). Podocytes cultured in only medium were considered blank. Furthermore, podocytes were randomly treated with medium; 100 ng/mL insulin‐like growth factor 1 (IGF‐1) (a PI3K‐AKT activator, 100‐11; Peprotech); or 100 ng/mL IGF‐1 + 80 µM PrA + 80 µM OA. After that, cell apoptosis assays and western blot analysis were carried out.

### Cell Count Kit 8 (CCK‐8) assay

The CCK8 Signalway Antibody LLC (SAB), CP002 assay was applied to assess podocyte proliferation. After digestion with 0.25% trypsin‐ethylenediaminetetraacetic acid (T1300‐100; Solarbio), fully differentiated podocytes were inoculated in a 96‐well culture plate at 5 × 10^3^ cells/well. Four gradient concentrations of C5b‐6 complex (0.1, 0.2, 0.4, 0.8 µg/mL) and NHS were put into different wells to induce podocyte injury, podocytes treated with medium were considered blank. After 0, 24, 48, and 72 h, a 100 µL mixture of CCK8 (CCK8: serum‐free medium = 1:10) was added to each well, followed by incubation for 1 h in a 5% CO_2_ incubator at 37°C. A microplate reader was used to detect the 450 nm absorption. An optimal concentration of sC5b‐9 complex was chosen for all following experiments. Likewise, CCK‐8 was also used to investigate the effects of PrA and OA at different concentrations on the cell viability of damaged podocytes, and an optimal concentration was chosen for further study.

### Quantitative real‐time polymerase chain reaction (qRT‐PCR)

After 24 h treatment of PrA and OA, TRIzol Reagent (1596‐026; Invitrogen) was utilized to extract the total RNA from treated podocytes. Following quantification, the integrity of isolated RNA was detected by electrophoresis with a 1% agarose gel. Subsequently, reverse transcriptase (#K1622; Fermentas) was utilized to synthesize complementary DNA, and qRT‐PCR reactions were conducted on the ABI Prism 7300 (ABI‐7300; ABI) with a SYBR Green PCR kit (#K0223; Thermo Fisher Scientific). After normalizing to glyceraldehyde 3‐phosphate dehydrogenase (GAPDH), the messenger RNA levels of several associated genes were analyzed by the $2^{-\Delta \Delta C_{t}}$ method. Primers were designed using the AlleleID software (PREMIER Biosoft) and the sequences are as follows: nephrin (5′‐GGACCCACACTACTACTC‐3′ and 5′‐CTCTCCACCTCGTCATAC‐3′), desmin (5′‐GTGGATGCAGCCACTCTAGC‐3′ and 5′‐TTAGCCGCGATGGTCTCATAC‐3′), podocin (5′‐TTGTTTCCTGGCTCCTTC‐3′ and 5′‐TGCCTTGGGACTACTTTC‐3′), CD2AP (5′‐AAGGAGAACTAAATGGGAGACGA‐3′ and 5′‐CCGTTTGATGGGCAAATTGTCA‐3′), Bcl2 (5′‐TGGGCATAGATGTGTCAGG‐3′ and 5′‐CCATATTCATCGCGTGGAG‐3′), Bax (5′‐TTGCTACAGGGTTTCATC‐3′ and 5′‐ATTGCTGTCCAGTTCATC‐3′), GAPDH (5′‐CTGCCCAGAACATCATCC‐3′ and 5′‐CTCAGATGCCTGCTTCAC‐3′). The PCR procedure was carried out as follows: 95°C for 10 min; 40 cycles of 95°C for 15 s, 60°C for 45 s (Hong et al., 2011).

### Western blot analysis

Treated podocytes were lysed in RIPA buffer (R0010; Solarbio), supplemented with protease and phophatase inhibitors, for 30 min at 4°C. After centrifugation for 10 min at 4°C, 12,000*g*, proteins were obtained and quantified by a BCA kit (PICPI23223; Thermo), followed by separation using 10% and 15% sodium dodecyl sulfate‐polyacrylamide gel electrophoresis gels (Jrdun Biotechnology Co., Ltd., Shanghai, China) and semi‐dry transferred onto polyvinylidene fluoride (PVDF) membranes (HATF00010; Millipore). The PVDF membranes containing target bands were blocked with 5% bovine serum albumin (BSA) in phosphate‐buffered saline with Tween 20 (PBST) for 1 h at room temperature, and then incubated with specific primary antibodies against nephrin (1:500, Sc‐377246; Santa Cruz Biotechnology), podocin (1:1,500, Ab50339; Abcam), CD2AP (1:1,000, #2135; Cell Signaling Technology [CST]), desmin (1:1,000, #4024; CST), Bcl2 (1:500, Sc‐492; Santa Cruz Biotechnology), Bax (1:500, Sc‐493; Santa Cruz Biotechnology), p‐AKT (1:1,000, #9271; CST), AKT (1:1,000, #9272; CST), p‐mTOR (1:1,000, #2974; CST), mTOR (1:1,000, #2972; CST), or GAPDH (1:2,000, #5174; CST) at 4°C overnight with gentle shaking. After 5–6 washes, the membranes were then incubated in secondary antibodies (1:5,000; Beyotime) for 2 h in the dark at room temperature. Membranes were again washed 5–6 times and incubated with chemiluminescence detection reagent in the dark for 5 min. The bands of target proteins were visualized through an ECL imaging system (Tanon, Shanghai, China).

### Flow cytometry (FCM) detection

Podocyte apoptosis was detected by FCM analysis (BD) with a double staining of annexin V‐fluorescein isothiocyanate (FITC) and propidium iodide (PI). After centrifugation, about 5–10 million cells were resuspended in 195 µL annexin V‐FITC binding buffer. Following a 15 min incubation with 5 µL annexin V‐FITC at 4°C in the dark, cells were incubated with 5 µL PI staining solution for 5 min at 4°C in the dark. A tube without annexin V‐FITC/PI was used as a negative control. Through FCM analysis, the rates of apoptotic cells were determined. Annexin V‐FITC was green in fluorescence and marked early apoptotic cells. PI was red in fluorescence. Annexin V^+^/PI^+^ indicated late apoptotic or necrotic cells, Annexin V­/PI^−^ indicated viable cells.

### Immunofluorescence

Injured podocytes cultured on glass coverslips were washed with 0.02 M PBS and then fixed in 4% paraformaldehyde (10010018; National Medicines Corporation Ltd.) for 30 min. Following 10 min permeabilization with 0.5% Triton X‐100 (T8200; Solarbio), cells were blocked in 1% BSA (A8010; Solarbio) for 1 h at room temperature. Subsequently, the cells were incubated in specific primary antibodies against sC5b‐9 (1:5,000 dilution; ab55811; Abcam), nephrin (1:200 dilution; sc‐377246; Santa Cruz Biotechnology), or podocin (1:1,000 dilution; ab50339; Abcam) at 4°C overnight in a humidifying box. Residual antibodies on the cells were removed by washing with PBST for three times. Next, the cells were incubated in secondary antibodies (1:1,000 dilution; Beyotime) and labeled with fluorescein for 2 h at room temperature. Finally, cells were sealed with mounting medium (P0126; Beyotime). A laser‐scanning microscope was used to take all of the images.

### Lactate dehydrogenase (LDH) activity detection

After modeling for inducing podocyte injury, the supernatants were collected. According to the instruction, the activity of LDH in supernatants after MAC deposition was detected by LDH Test Kit (Nanjing Institute of Bioengineering, Nanjing, China).

### Statistical analysis

All statistical analyses in this paper were carried by GraphPad prism 7.0. Student's *t* test was used to compare the two groups, while multiple comparisons were analyzed using one‐way analysis of variance followed by Tukey's multiple comparisons. All data values were obtained from at least three independent experiments and shown as mean ± standard deviation. The *P* < 0.05 is considered statistically significant.

## Results

### Successful modeling of podocyte injury induced by the sC5b‐9 complex

**Figure 1:**
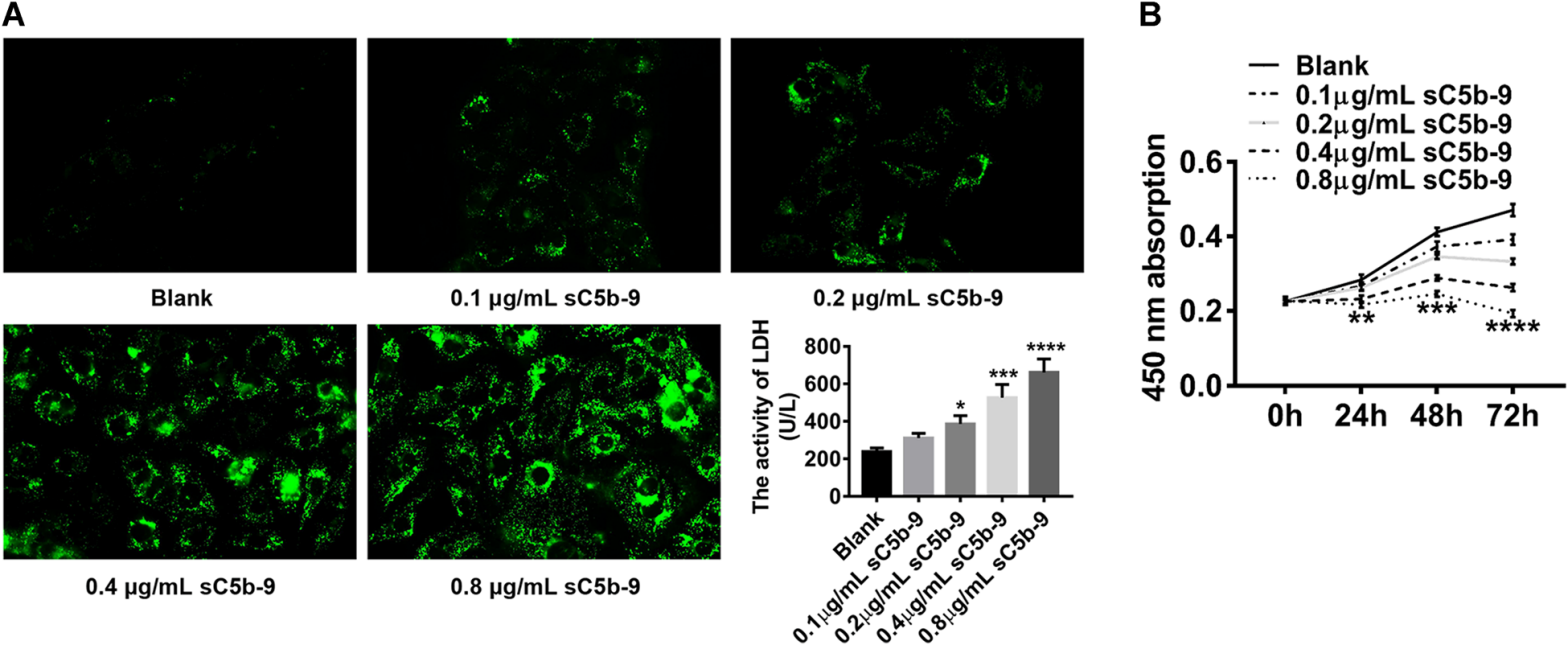
**Successful modeling of podocyte injury induced by the C5b‐9 complex.** After 0, 24, 48, and 72 h treatment of C5b‐6 complex and normal human serum (NHS) at four gradient concentrations (0.1, 0.2, 0.4, 0.8 µg/mL), cells were stained for immunofluorescence and lactate dehydrogenase (LDH) activity in supernatants was detected (A; magnification: ×400), and cell viability of podocytes at 450 nm was measured by a cell counting kit‐8 (CCK‐8) assay (B). Blank: podocytes treated with only medium. Data shown are representative of at least three independent experiments, data are expressed as mean ± standard deviation (*P < 0.05; **P < 0.01; ***P < 0.001; ****P < 0.0001 compared with blank).

After treatment of different concentrations of C5b‐6 complex and NHS, the cell viability of podocytes was evaluated using a CCK8 assay at 0, 24, 48, and 72 h. As shown in Figure 1A, when sC5b‐9 < 0.8 µg/mL, immunofluorescence showed that the deposition of sC5b‐9 on the surface of podocytes smaller increased, and the activity of LDH in the culture supernatant was low, indicating that the cell lysis was very small at this time. When sC5b‐9 = 0.8 µg/mL, sC5b‐9 deposition on podocytes significantly increased, and LDH activity in cells also increased significantly, indicating the higher activity of MAC in the system. Furthermore, the sC5b‐9 complex had a significant inhibitory effect on podocyte proliferation in a dose‐ and time‐dependent manner. The cell viability of podocytes treated with 0.8 µg/mL sC5b‐9 was remarkably lower than that of cells treated with other concentrations of sC5b‐9 (Figure 1B). Therefore, it preliminarily suggested that an in vitro model of podocyte injury was successfully induced by C5b‐6 and NHS, and an optimal concentration of 0.8 µg/mL sC5b‐9 was chosen for further study (Chen et al., 2010).

### The effects of sC5b‐9 complex on podocyte apoptosis and several associated genes

**Figure 2:**
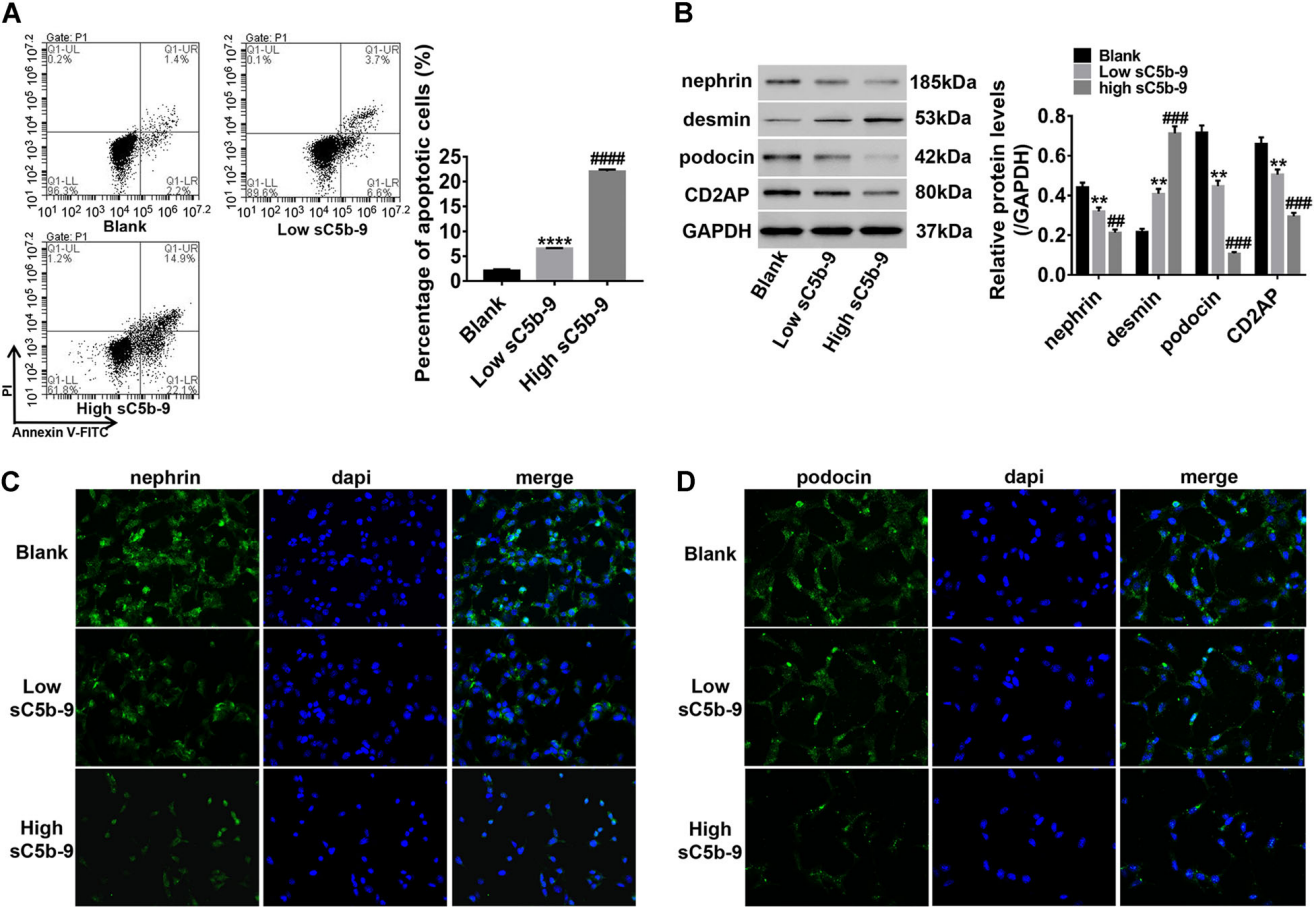
**The effects of the C5b‐9 complex on podocyte apoptosis and several associated genes.** Successful modeling with 0.1 and 0.8 µg/mL C5b‐6 were chosen to continually treat with a normal medium for 48 h. Apoptotic cells were analyzed by flow cytometry (FCM) (A) while western blot analysis was performed to quantify the protein levels of associated genes (B). Lower right quadrant shows early apoptotic cells stained with annexin‐V; upper right quadrant represents late apoptotic or necrotic cells that were stained double positive with two dyes annexin‐V and propidium iodide (PI); lower left quadrant represents viable cells. Immunofluorescence staining of nephrin (C) and podocin (D) was performed (magnification: ×400). Blank: podocytes treated with the only medium. Data shown are representative of at least three independent experiments. All data are shown as mean ± standard deviation (**P and ^##^ *P* < 0.01; ^###^ *P* < 0.001^; ****^ *P* and ^####^ *P* < 0.0001 compared with blank).

Cells in the blank, low sC5b‐9 (0.1 µg/mL), and high sC5b‐9 (0.8 µg/mL) groups were used to explore the effect of the sC5b‐9 complex on podocyte apoptosis and several associated genes. FCM analysis revealed that the sC5b‐9 complex induced early apoptosis of podocyte, and its induction of apoptosis was enhanced with increasing concentrations (Figure 2A). The protein levels of nephrin, podocin, and CD2AP were significantly reduced by the sC5b‐9 complex, while desmin was elevated (Figure 2B). Moreover, immunofluorescence of nephrin and podocin further showed the inhibitory effects of the sC5b‐9 complex on nephrin (Figure 2C) and podocin (Figure 2D) expression. All data suggested that the sC5b‐9 complex injured podocytes by promoting apoptosis and regulating the expression of associated genes.

### PrA and OA have a protective effect on podocyte apoptosis induced by podocyte injury

**Figure 3:**
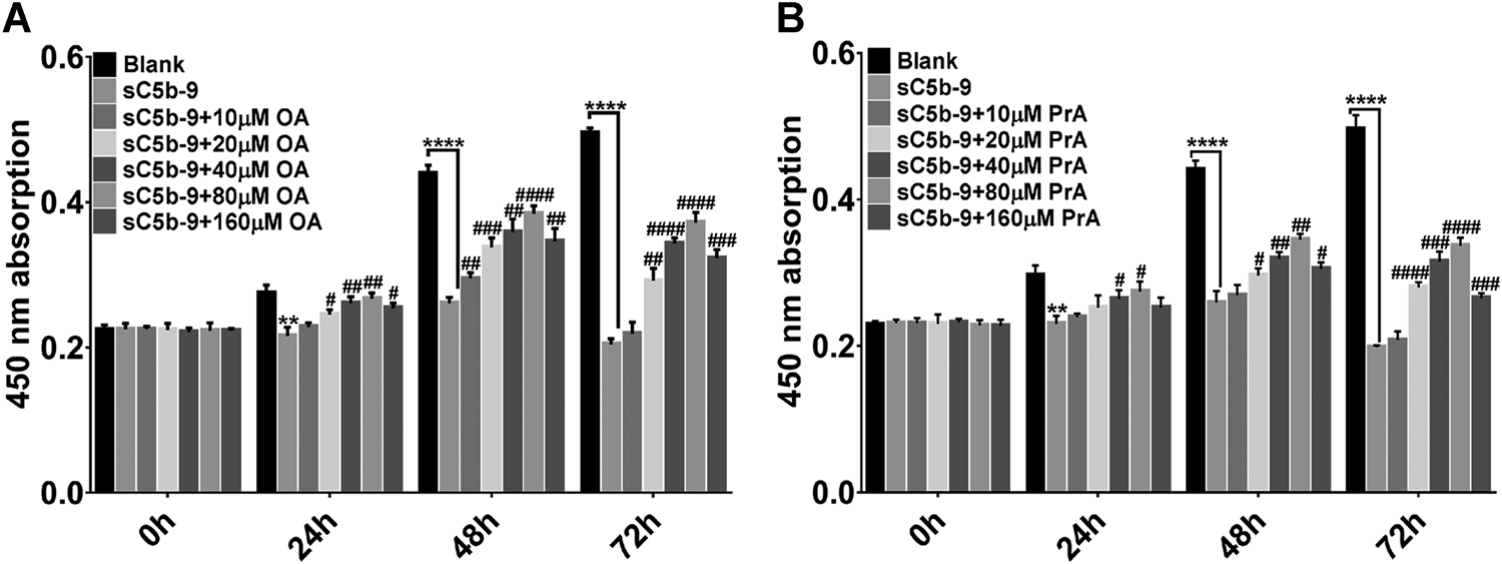
**Protosappanin‐A (PrA) and oleanolic acid (OA) has a protective effect on podocyte apoptosis induced by podocyte injury.** After treated with different concentrations of PrA (0, 10, 20, 40, 80, 160 µM) and OA (0, 10, 20, 40, 80, 160 µM) for 0, 24, 48, and 72 h, the cell proliferation of injured podocytes was detected by a cell counting kit‐8 (CCK‐8) assay; podocytes treated with only medium are considered as blank. Data are representative of at least three independent experiments and results are shown as mean ± standard deviation (SD) (**P < 0.01,****P < 0.0001 compared with blank; ^#^ *P* < 0.05, ^##^ *P* < 0.01, ^###^ *P* < 0.001, ^####^ *P* < 0.0001 compared with sC5b‐9 [0.8 µg/mL]).

After 0, 24, 48, and 72 h treatment of PrA and OA, the cell proliferation of injured podocytes was detected by a CCK8 assay. As shown in Figure 3, both PrA (Figure 3A) and OA (Figure 3B) significantly promoted the cell proliferation of injured podocytes in a dose‐ and time‐dependent manner and reached their peak effects at 80 µM. The same dose of OA showed a stronger protective effect than that of PrA. These results indicated that there was a protective effect of PrA and OA on podocyte injury through inhibition of podocyte apoptosis. An optimal concentration of 80 µM was chosen for further study.

### PrA and OA protect injured podocytes from apoptosis through the regulation of several associated genes and changes in signaling

**Figure 4:**
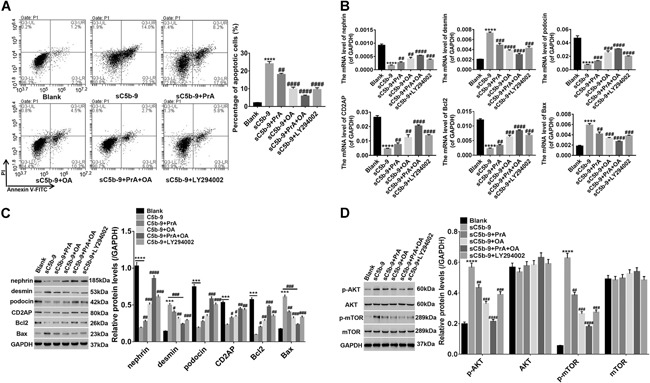
**Protosappanin‐A (PrA) and oleanolic acid (OA) protect injured podocytes from apoptosis through the regulation of several associated genes and changes in signaling.** Injured podocytes were respectively treated with medium, 80 µM PrA, 80 µM OA, 80 µM combined PrA with OA or 5 µM LY294002. After 24 h, podocyte apoptosis was analyzed by flow cytometry (FCM) (A). Lower right quadrant shows early apoptotic cells stained with annexin‐V; upper right quadrant represents late apoptotic or necrotic cells that were stained double‐positive for two dyes annexin‐V and propidium iodide (PI); lower left quadrant represents viable cells. The messenger RNA (mRNA) (B) levels of several associated genes were quantified by quantitative real‐time polymerase chain reaction (qRT‐PCR) while the protein levels of genes (C) and changes in signaling (D) were quantified by western blot analysis. Blank: podocytes treated with only medium. Data shown are representative of at least three independent experiments, all data are shown as mean ± standard deviation (***P < 0.001, ****P < 0.0001 compared with blank; ^#^ *P* < 0.05, ^##^ *P* < 0.01, ^###^ *P* < 0.001, ^####^ *P* < 0.0001 compared with sC5b‐9).

Injured podocytes were treated, either alone or in combination, with PrA and OA. Early apoptotic cells of injured podocytes were obviously reduced by PrA and OA (Figure 4A), accompanied by markedly increased levels of nephrin, podocin, CD2AP, and B‐cell lymphoma 2 (Bcl2) along with decreased levels of desmin and Bcl‐2‐associated X proteins (Bax). The effects of the combination of the two drugs were significantly better than treatment with any single drug (Figures 4B and 4C). Studies have reported that Bcl2 is an anti‐apoptotic protein in cancers, whereas Bax has a pro‐apoptotic effect, and the radio of Bax to Bcl‐2 is used to determine whether the cells will commit to apoptosis (Green and Reed, 1998; Gao and Dou, 2000). Further, C5b‐9‐induced increase of p‐AKT (Ser473)/p‐mTOR (Ser2481) levels in damaged podocytes was also remarkably reversed by PrA and OA, while the levels of AKT and mTOR remained unchanged (Figure 4D). These results were also consistent with the effects of the PI3K/AKT inhibitor LY294002 on injured podocytes in our study. Therefore, we conjectured that podocyte injury was likely to cause abnormal activation of AKT/mTOR signaling, and PrA and OA protected injured podocytes from apoptosis mainly through inhibiting phosphorylation of AKT/mTOR and the regulation of expression in associated genes.

### PrA and OA suppress podocyte apoptosis by inhibiting the AKT/mTOR signaling pathway

**Figure 5:**
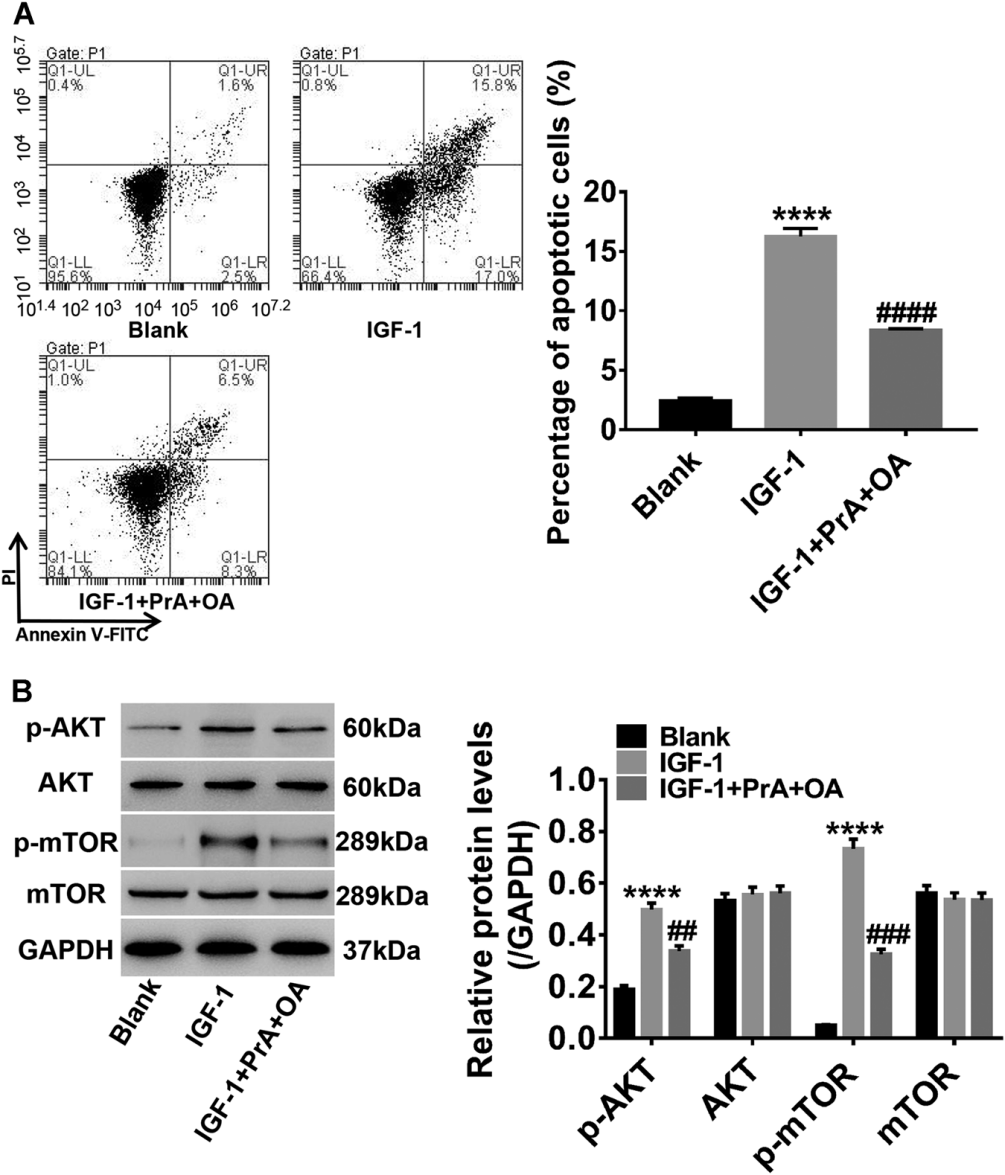
**Protosappanin‐A (PrA) and oleanolic acid (OA) suppress podocyte apoptosis by inhibiting AKT/mTOR signaling.** After 24 h treatment with 100 ng/mL insulin‐like growth factor 1 (IGF‐1) (a PI3K‐AKT activator) and IGF + 80 µM PrA + 80 µM OA, FCM analysis was carried out to assess podocyte apoptosis (A). Lower right quadrant shows early apoptotic cells stained with annexin‐V; upper right quadrant represents late apoptotic or necrotic cells that were stained double‐positive for two dyes annexin‐V and propidium iodide (PI); lower left quadrant represents viable cells. AKT/mTOR signaling levels were quantified using western blot analysis (B). Blank: podocytes treated with only medium. Data shown are representative of at least three independent experiments, all data are shown as mean ± standard deviation (****P < 0.0001 compared with blank; ^##^ *P* < 0.01, ^###^ *P* < 0.001, ^####^ *P* < 0.0001 compared with IGF‐1).

Podocytes were treated with IGF‐1 (a PI3K‐AKT activator) and PrA combined with OA. As shown in Figure 5, a significant increase of early apoptosis in podocytes was detected after IGF‐1 treatment, and IGF‐1‐induced apoptosis was remarkably inhibited by PrA and OA (Figure 5A). Furthermore, p‐AKT/p‐mTOR levels in podocytes were significantly increased by IGF‐1 while levels of AKT and mTOR were unaltered. Increases in p‐AKT/p‐mTOR levels were also markedly reversed by PrA and OA (Figure 5B). These results further demonstrate that inhibition of podocyte apoptosis by PrA and OA may be via inhibition of AKT/mTOR signaling.

## Discussion

Podocytes, a size‐selective filtration barrier in the glomerulus, are injured in a great number of glomerular diseases. Several effective monomers isolated from *C. sappan* L. have been reported to exhibit therapeutic potential on these diseases. Ursolic acid is reported to improve podocyte injury (Xu et al., 2017). In addition, OA and *N*‐acetylcysteine can ameliorate the progression of DN (Lee et al., 2016). Here, we aimed to investigate the effects as well as the mechanism of PrA and OA on podocyte injury.

We found that sC5b‐9‐induced apoptosis was significantly inhibited by the treatment of OA and PrA, and the combination of two drugs was significantly more efficacious than treatment with single drug. Treatment with drug was accompanied by a decrease in desmin level as well as the restoration of nephrin, podocin, and CD2AP. Nephrin, podocin, CD2AP, and desmin are important components of SD functioning in podocyte processes (Ruotsalainen et al., 1999; Huber et al., 2001; Schwarz et al., 2001; Shih et al., 2001; Roselli et al., 2002). Thus, we concluded that OA and PrA could protect podocytes from C5b‐9 induced‐injury by regulating nephrin, podocin, and CD2AP, and desmin expression.

AKT, a major downstream mediator of PI3K activity, was found to be required for the survival of various cells and blocked toxic stimuli‐induced apoptosis. PI3K‐AKT could control the regulation of gene expression, cell viability, growth, and other complex cellular programs (Cantley, 2002; Edinger and Thompson, 2002; Shioi et al., 2002). It was reported that Akt is activated in OA‐treated vascular smooth muscle cells (VSMCs), which may be involved in OA‐induced heme oxygenase‐1 (HO‐1) expression in VSMCs (Feng et al., 2011). Moreover, notoginsenoside R1 (NR1), the major component of a Chinese herbal medicine, was reported to ameliorate podocyte injury through activation of PI3K/AKT signaling (Huang et al., 2016). In our study, increased p‐AKT/p‐mTOR levels in damaged podocytes were remarkably reduced by PrA and OA. PI3K/AKT inhibitor LY294002 showed similar protective effects on injured podocytes as OA and PrA. Interaction between nephrin, podocin, CD2AP, and PI3K activated AKT signaling is reported to protect podocytes from injury or apoptosis (Huber et al., 2003; Chuang and He, 2009). Regulation of mTOR‐ULK1 signaling is also correlated with improvement after podocyte injury (Lingling, 2013; Wu et al., 2013). Taken together, we inferred that OA and PrA protected podocytes from apoptosis or injury by inhibiting the abnormal activation of PI3K‐AKT‐mTOR signaling. Our conclusion is further supported by the observation that the effects of induced PI3K/AKT by IGF‐1 on podocyte apoptosis and p‐AKT/p‐mTOR levels was reversed by OA and PrA. One limitation is that the model for inducing podocyte injury was not very good with low apoptosis levels. However, compared with normal podocytes, the injury model still caused a certain degree of apoptosis, and the subsequent experiments also changed significantly. Therefore, our research results still have certain significance. If possible, we will further improve our model for podocyte injury to make our conclusions more convincing.

## Conclusion

This paper found protective effects of OA and PrA on damaged podocytes in terms of cell apoptosis. In addition, a combination of the two drugs resulted in an additive effect, and may be through inhibition of the abnormal activation of the PI3K‐AKT‐mTOR signaling pathway. These results reveal a novel promising therapy for improvement and treatment of podocyte injury in glomerular diseases.

## Acknowledgments and funding

This work was funded by the Heilongjiang Nature Science Foundation (key project) (Grant No. ZD2017018).

## Conflict of interest

All authors declare that they have no conflict of interest.

## Ethical approval

All procedures performed in studies involving humans were in accordance with the ethical standards of the institution or practice at which the study was conducted.

## References

[cbin11218-bib-0001] Aleshin AE , DiScipio RG , Stec B , Liddington RC (2012) Crystal structure of C5b‐6 suggests structural basis for priming assembly of the membrane attack complex. J Biol Chem 287(23): 19642–52, 10.1074/jbc.M112.361121 22500023 PMC3365999

[cbin11218-bib-0002] Badami S , Moorkoth S , Rai SR , Kannan E , Bhojraj S (2003) Antioxidant activity of *Caesalpinia sappan* heartwood. Biol Pharm Bull 26(11): 1534–7. 14600396 10.1248/bpb.26.1534

[cbin11218-bib-0003] Baek NI , Jeon SG , Ahn EM , Hahn JT , Bahn JH , Jang JS , Cho SW , Park JK , Choi SY (2000) Anticonvulsant compounds from the wood of *Caesalpinia sappan* L. Arch Pharm Res 23(4): 344–8. 10976581 10.1007/BF02975445

[cbin11218-bib-0004] Boute N , Gribouval O , Roselli S , Benessy F , Lee H , Fuchshuber A , Antignac C (2000) Correction to “NPHS2, encoding the glomerular protein podocin, is mutated in autosomal recessive steroid‐resistant nephrotic syndrome”. Nat Genet 25(1): 125. 10742096 10.1038/74166

[cbin11218-bib-0005] Cantley LC (2002) The phosphoinositide 3‐kinase pathway. Science 296(5573): 1655–7. 12040186 10.1126/science.296.5573.1655

[cbin11218-bib-0006] Chen Z‐H , Qin W‐S , Zeng C‐H , Zheng C‐X , Hong Y‐M , Lu Y‐Z , Li LS , Liu ZH (2010) Triptolide reduces proteinuria in experimental membranous nephropathy and protects against C5b‐9‐induced podocyte injury in vitro. Kidney Int 77(11): 974–88, 10.1038/ki.2010.41 20375980

[cbin11218-bib-0007] Chuang PY , He JC (2009) Signaling in regulation of podocyte phenotypes. Nephron Physiol 111(2): p9–15. 19142027 10.1159/000191075 PMC2881215

[cbin11218-bib-0008] Dubey VK , Patil CR , Kamble SM , Tidke PS , Patil KR , Maniya PJ , Jadhav RB , Patil SP (2013) Oleanolic acid prevents progression of streptozotocin induced diabetic nephropathy and protects renal microstructures in Sprague Dawley rats. J Pharmacol Pharmacother 4(1): 47–52. 23662024 10.4103/0976-500X.107678 PMC3643343

[cbin11218-bib-0009] Durvasula RV , Shankland SJ (2006) Podocyte injury and targeting therapy: an update. Curr Opin Nephrol Hypertens 15(1): 1–7. 16340659 10.1097/01.mnh.0000199012.79670.0b

[cbin11218-bib-0010] Edinger, AL , Thompson CB (2002) Akt maintains cell size and survival by increasing mTOR‐dependent nutrient uptake. Mol Biol Cell 13(7): 2276–88. 12134068 10.1091/mbc.01-12-0584 PMC117312

[cbin11218-bib-0011] Feng J , Zhang P , Chen X , He G (2011) PI3K and ERK/Nrf2 pathways are involved in oleanolic acid‐induced heme oxygenase‐1 expression in rat vascular smooth muscle cells. J Cell Biochem 112(6): 1524–31. 21328610 10.1002/jcb.23065

[cbin11218-bib-0012] Floege J , Hackmann B , Kliem V , Kriz W , Alpers CE , Johnson RJ , Kühn KW , Koch KM , Brunkhorst R (1997) Age‐related glomerulosclerosis and interstitial fibrosis in Milan normotensive rats: a podocyte disease. Kidney Int 51(1): 230–43. 8995738 10.1038/ki.1997.28

[cbin11218-bib-0013] Gao G , Dou QP (2000) G1 phase‐dependent expression of Bcl‐2 mRNA and protein correlates with chemoresistance of human cancer cells. Mol Pharmacol 58(5): 1001–10. 11040047 10.1124/mol.58.5.1001

[cbin11218-bib-0014] Green DR , Reed JC (1998) Mitochondria and apoptosis. Science 281(5381): 1309–12. 9721092 10.1126/science.281.5381.1309

[cbin11218-bib-0015] Guan N , Ding J , Zhang JJ (2004) Relationship between expression of nephrin, podocin and α‐actinin‐4 with proteinuria in puromycin aminonucleoside nephrosis rats. Chin J Nephrol 20: 26–32.

[cbin11218-bib-0016] Hadders MA , Bubeck D , Roversi P , Hakobyan S , Forneris F , Morgan BP , Pangburn MK , Llorca O , Lea SM , Gros P (2012) Assembly and regulation of the membrane attack complex based on structures of C5b6 and sC5b9. Cell Rep 1(3): 200–7, 10.1016/j.celrep.2012.02.003 22832194 PMC3314296

[cbin11218-bib-0017] Hemmelgarn, BR (2010) Relation between kidney function, proteinuria, and adverse outcomes. JAMA 303(5): 423. 20124537 10.1001/jama.2010.39

[cbin11218-bib-0018] Hong J , Kang B , Kim A , Hwang S , Ahn J , Lee S , Kim J , Park JH , Cheon DS (2011) Development of a highly sensitive real‐time one step RT‐PCR combined complementary locked primer technology and conjugated minor groove binder probe. Virol J 8(1): 330. 21714898 10.1186/1743-422X-8-330 PMC3142241

[cbin11218-bib-0019] Hong SY , Yu F‐X , Luo Y , Hagen T (2016) Oncogenic activation of the PI3K/Akt pathway promotes cellular glucose uptake by downregulating the expression of thioredoxin‐interacting protein. Cell Signal 28(5): 377–83. 26826652 10.1016/j.cellsig.2016.01.011

[cbin11218-bib-0020] Hoshi S , Shu Y , Yoshida F , Inagaki T , Sonoda J , Watanabe T , Nomoto K , Nagata M (2002) Podocyte injury promotes progressive nephropathy in zucker diabetic fatty rats. Lab Invest 82(1): 25–35. 11796823 10.1038/labinvest.3780392

[cbin11218-bib-0021] Huang G , Lv J , Li T , Huai G , Li X , Xiang S , Wang L , Qin Z , Pang J , Zou B , Wang Y (2016) Notoginsenoside R1 ameliorates podocyte injury in rats with diabetic nephropathy by activating the PI3K/Akt signaling pathway. Int J Mol Med 38(4): 1179–89. 27571993 10.3892/ijmm.2016.2713 PMC5029967

[cbin11218-bib-0022] Huber TB , Hartleben B , Kim J , Schmidts M , Schermer B , Keil A , Egger L , Lecha RL , Borner C , Pavenstädt H , Shaw AS , Walz G , Benzing T (2003) Nephrin and CD2AP associate with phosphoinositide 3‐OH kinase and stimulate AKT‐dependent signaling. Mol Cell Biol 23(14): 4917–28. 12832477 10.1128/MCB.23.14.4917-4928.2003 PMC162232

[cbin11218-bib-0023] Huber TB , Köttgen M , Schilling B , Walz G , Benzing T (2001) Interaction with podocin facilitates nephrin signaling. J Biol Chem 276(45): 41543–6. 11562357 10.1074/jbc.C100452200

[cbin11218-bib-0024] Kang HG , Paik KH , Cho HY , Lee BH , Ha IS , Choi Y , Cheong HI (2010) Transcriptome analysis of the response of cultured murine podocytes to puromycin aminonucleoside. Nephron Exp Nephrol 115(1): e1–8. 20185935 10.1159/000286518

[cbin11218-bib-0025] Kim EC , Hwang YS , Lee HJ , Lee SK , Park MH , Jeon BH , Jeon CD , Lee SK , Yu HH , You YO (2005) Caesalpinia sappan induces cell death by increasing the expression of p53 and p21WAF1/CIP1 in head and neck cancer cells. Am J Chin Med 33(03): 405–14. 16047558 10.1142/S0192415X05003016

[cbin11218-bib-0026] Kim JM (2003) CD2‐associated protein haploinsufficiency is linked to glomerular disease susceptibility. Science 300(5623): 1298–300. 12764198 10.1126/science.1081068

[cbin11218-bib-0027] Lee ES , Kim HM , Kang JS , Lee EY , Yadav D , Kwon MH , Kim YM , Kim HS , Chung CH (2016) Oleanolic acid and *N*‐acetylcysteine ameliorate diabetic nephropathy through reduction of oxidative stress and endoplasmic reticulum stress in a type 2 diabetic rat model. Nephrol Dial Transplant 31(3): 391–400. 26567248 10.1093/ndt/gfv377

[cbin11218-bib-0028] Li C , Ruotsalainen V , Tryggvason K , Shaw AS , Miner JH (2000) CD2AP is expressed with nephrin in developing podocytes and is found widely in mature kidney and elsewhere. Am J Physiol Renal Physiol 279(279): F785–792. 10997929 10.1152/ajprenal.2000.279.4.F785

[cbin11218-bib-0029] Lingling W (2013) Rapamycin reduced podocyte injury by inhibiting the mTOR‐ULK1 signaling pathway. PLoS One 8: e63799. 23667674 10.1371/journal.pone.0063799 PMC3648526

[cbin11218-bib-0030] Liu J (1995) Pharmacology of oleanolic acid and ursolic acid. J Ethnopharmacol 49(2): 57–68. 8847885 10.1016/0378-8741(95)90032-2

[cbin11218-bib-0031] Ma H , Sandor DG , Beck LH (2013) The role of complement in membranous nephropathy. Semin Nephrol 33(6): 531–42, 10.1016/j.semnephrol.2013.08.004 24161038 PMC4274996

[cbin11218-bib-0032] Machado JR , Rocha LP , Neves PD , Cobô Ede C , Silva MV , Castellano LR , Corrêa RR , Reis MA (2012) An overview of molecular mechanism of nephrotic syndrome. Int J Nephrol 2012: 937623. 22844593 10.1155/2012/937623 PMC3401527

[cbin11218-bib-0033] Patrakka J , Kestilä M , Wartiovaara J , Ruotsalainen V , Tissari P , Lenkkeri U , Männikkö M , Visapää I , Holmberg C , Rapola J , Tryggvason K , Jalanko H (2000) Congenital nephrotic syndrome (NPHS1): features resulting from different mutations in Finnish patients. Kidney Int 58(3): 972–80. 10972661 10.1046/j.1523-1755.2000.00254.x

[cbin11218-bib-0034] Pollier J , Goossens A (2012) Oleanolic acid. Phytochemistry 77: 10–5. 22377690 10.1016/j.phytochem.2011.12.022

[cbin11218-bib-0035] Roselli S , Gribouval O , Boute N , Sich M , Benessy F , Attié T , Gubler MC , Antignac C (2002) Podocin localizes in the kidney to the slit diaphragm area. Am J Pathol 160(1): 131–9. 11786407 10.1016/S0002-9440(10)64357-X PMC1867125

[cbin11218-bib-0036] Schwarz K , Simons M , Reiser J , Saleem MA , Faul C , Kriz W , Shaw AS , Holzman LB , Mundel P (2001) Podocin, a raft‐associated component of the glomerular slit diaphragm, interacts with CD2AP and nephrin. J Clin Invest 108(11): 1621–9. 11733557 10.1172/JCI12849 PMC200981

[cbin11218-bib-0037] Shankland SJ , Al'Douahji M (1999) Cell cycle regulatory proteins in glomerular disease. Exp Nephrol 7(3): 207–11. 10352360 10.1159/000020603

[cbin11218-bib-0038] Shankland SJ (2006) The podocyte's response to injury: role in proteinuria and glomerulosclerosis. Kidney Int 69(12): 2131–47. 16688120 10.1038/sj.ki.5000410

[cbin11218-bib-0039] Shih NY , Li J , Cotran R , Mundel P , Miner JH , Shaw AS (2001) CD2AP localizes to the slit diaphragm and binds to nephrin via a novel C‐terminal domain. Am J Pathol 159(6): 2303–8. 11733379 10.1016/S0002-9440(10)63080-5 PMC1850607

[cbin11218-bib-0040] Shioi T , Mcmullen JR , Kang PM , Douglas PS , Obata T , Franke TF , Cantley LC , Izumo S (2002) Akt/protein kinase B promotes organ growth in transgenic mice. Mol Cell Biol 22(8): 2799–809. 11909972 10.1128/MCB.22.8.2799-2809.2002 PMC133704

[cbin11218-bib-0041] Ruotsalainen V , Ljungberg P , Wartiovaara J , Lenkkeri U , Kestila M , Jalanko H , Holmberg C , Tryggvason K (1999) Nephrin is specifically located at the slit diaphragm of glomerular podocytes. Proc Natl Acad Sci USA 96(14): 7962–7. 10393930 10.1073/pnas.96.14.7962 PMC22170

[cbin11218-bib-0042] Wang D , Dai C , Li Y , Liu Y (2011) Canonical Wnt/β‐catenin signaling mediates transforming growth factor‐β1‐driven podocyte injury and proteinuria. Kidney Int 80(11): 1159–69. 21832980 10.1038/ki.2011.255 PMC4300105

[cbin11218-bib-0043] Wei M , Li Z , Xiao L , Yang Z (2015) Effects of ROS‐relative NF‐κB signaling on high glucose‐induced TLR4 and MCP‐1 expression in podocyte injury. Mol Immunol 68(2 Pt A): 261–71. 26364141 10.1016/j.molimm.2015.09.002

[cbin11218-bib-0044] Wolf G , Stahl RA (2003) CD2‐associated protein and glomerular disease. Lancet 362(9397): 1746–8. 14643126 10.1016/S0140-6736(03)14856-8

[cbin11218-bib-0045] Wu J , Hou JB , Zhang MM , Zou YP , Yu B (2008) Protosappanin a, an immunosuppressive constituent from a Chinese herb, prolongs graft survival and attenuates acute rejection in rat heart allografts. Transpl Proc 40(10): 3719–22. 10.1016/j.transproceed.2008.06.097 19100473

[cbin11218-bib-0046] Wu J , Zhang M , Jia H , Huang X , Zhang Q , Hou J , Bo Y (2010) Protosappanin A induces immunosuppression of rats heart transplantation targeting T cells in grafts via NF‐κB pathway. Naunyn chmiedebergs Arch Pharmacol 381(1): 83–92. 10.1007/s00210-009-0461-5 19924402

[cbin11218-bib-0047] Wu L , Feng Z , Cui S , Hou K , Tang L , Zhou J , Cai G , Xie Y , Hong Q , Fu B , Chen X (2013) Rapamycin upregulates autophagy by inhibiting the mTOR‐ULK1 pathway, resulting in reduced podocyte injury. PLoS One 8(5): e63799. 23667674 10.1371/journal.pone.0063799 PMC3648526

[cbin11218-bib-0048] Xu L , Fan Q , Wang X , Li L , Lu X , Yue Y , Cao X , Liu J , Zhao X , Wang L (2017) Ursolic acid improves podocyte injury caused by high glucose. Nephrol Dial Transplant 32(8): 1285–93. 26567247 10.1093/ndt/gfv382

[cbin11218-bib-0049] Zhang H‐T , Wang W‐W , Ren L‐H , Zhao X‐X , Wang Z‐H , Zhuang D‐L , Bai Y‐N (2016) The mTORC2/Akt/NFκB pathway‐mediated activation of TRPC6 participates in adriamycin‐induced podocyte apoptosis. Cell Physiol Biochem 40(5): 1079–93. 27960162 10.1159/000453163

